# Oral health-related quality of life among a group of patients with substance use disorders in rehabilitation treatment: a cross-sectional study

**DOI:** 10.1186/s12903-021-01764-0

**Published:** 2021-08-19

**Authors:** Saeed Amiri, Hajar Shekarchizadeh

**Affiliations:** 1 School of Dentistry, Isfahan (Khorasgan) Branch, Islamic Azad University, University Blvd., Arghavanieh, East Jey St., Isfahan, Iran; 2 Community Health Research Center, Department of Community Oral Health, School of Dentistry, Isfahan (Khorasgan) Branch, Islamic Azad University, University Blvd., Arghavanieh, East Jey St, P.O. Box: 81595-158, Isfahan, Iran

**Keywords:** Illicit drugs, Oral health, Dental caries, Quality of life

## Abstract

**Background:**

Little is known about the effect of illicit drugs on oral health-related quality of life. Our aim was to investigate oral health-related quality among patients with substance use disorders, and its association with dental caries experience and drug use profile.

**Methods:**

Applying a stratified cluster random sampling method, we conducted a cross-sectional study on 267 in-treatment patients with substance use disorders in Isfahan, Iran in 2016. Self-administered questionnaires requested participants’ demographics and drug use profile. A trained dental student carried out personal interviews utilizing Oral Impact on Daily Performance (OIDP) instrument. Clinical examinations were conducted to record dental caries experience. *T* test, ANOVA, pearson and spearman correlation coefficient, and linear regression model served for statistical analysis (*p* < 0.05).

**Results:**

A great majority of the participants reported past use of opium (85%) followed by heroin (42.7%) and amphetamines (20.2%). The most common routes of drug administration were combined routes (44.6%) followed by smoking (36.7%). The mean score of OIDP was 22.4 ± 8.6. As high as 74.1% of the participants reported at least one OIDP impact. The most prevalent OIDP impact was “difficulty eating” (64.8%). The most prevalent cause of the impacts were “dental decay” and “tooth loss”. No significant association revealed between OIDP and patients’ demographics and drug use profile (*p* > 0.05). Participants with higher caries experience, reported greater OIDP (*p* < 0.05).

**Conclusions:**

There is an oral impact on the daily performance of patients with substance use disorders. Patients with higher caries experience reported greater OIDP. Thus, in addition to normative assessment of oral health, clinicians should consider the patients’ self-reported oral problems, and the social and mental aspects of oral conditions.

## Background

According to the world drug report 2019, 271 million people (5.5% of the world population) aged 15–64 in 2017, reported using drugs during the previous year among which 35 million people suffered from drug use disorders [1]. Among various health problems associated with drug addiction, oral health problems are very serious and prevalent, thus require more attention not only by dentists but also by clinicians, addiction care providers and policymakers [2].

Oral complications associated with drugs include tooth loss, generalized dental decay, periodontal diseases, oral mucosal diseases, candidiasis, mucosal dysplasia, xerostomia, erosion, bruxism, jaw clenching, tooth wear, and temporomandibular disorders [2, 3]. These problems may be the direct consequences of drugs on oral cavity, or may indirectly result from drug adverse effects such as chaotic life style, carelessness, traumatic orofacial injuries, poor oral hygiene, increased carbohydrate intake in diet, and inappropriate nutrition. In addition, low priority of oral health and low use of dental services might complicate the situation [2].

Some studies have shown the effect of oral problems on quality of life of patients with substance use disorders (SUD) [4–8]. As a subjective assessment of oral health, this socio-dental indicator describes how oral complications disrupt individual’s normal social function [9]. Normative assessments by objective measures do not reflect perceived health or perceived needs. Thus, oral health professionals should also consider the impact of oral problems on patients’ quality of life as part of their oral health need assessment [10, 11]. In addition, understanding the social impact of oral complications helps policy maker to prioritize the resources and to reduce the inequalities [12].

Among a variety of instruments assessing oral health-related quality of life, the Oral Impacts on Daily Performances (OIDP) is a commonly used, relatively brief and theoretically sound instrument that measures the impacts of oral conditions on individuals’ daily activities and behaviours [10, 13].

Although, scientific evidence about adverse effects of illicit drugs on oral health are growing [2, 3, 14], few published data exist regarding oral health-related quality of life (OHRQoL) of patients with SUD. A study by Marques reported the effect of dental caries on quality of life of patients with SUD [4]. In another study, injecting drug users showed poorer OHRQoL of life than did general population [6]. Among US Methamphetamine users, both oral health and OHRQoL reported to be worse than among general population [5].

Since the impact of illicit drugs on OHRQoL has not established well, the aim of the present study was to investigate OHRQoL among patients with SUD attending outpatient treatment centers in Iran, and its association with their caries experience and their drug use profile.

## Methods

Applying a stratified cluster random sampling method, we conducted a cross-sectional study on 267 patients with SUD attending methadone maintenance treatment centers in Isfahan, Iran in 2016. First, we stratified all treatment centers into the private (n = 200) and public centers (n = 5). In terms of the patient’s costs, private centers receive more monthly fee from their clients than do public centers. Thus, more deprived patients usually tend to attend public centers. In addition, in Isfahan, people from different socioeconomic backgrounds are spread around the city. Thus, we categorized all 200 private centers based on five geographic areas of the city (North West, North East, Center, South west and South East). Then, in order to cover all geographic regions, from each region one private center was randomly selected as a cluster. We also randomly selected two clusters from the five existing public centers.

Target population comprised patients with SUD who met the DSM-IV criteria for opioid dependence [15], receiving methadone maintenance treatment in rehabilitation centers. Applying the proportional sampling, the minimum sample size of 250 was estimated. In total, 152 volunteer patients with SUD from private center and 115 volunteers from public centers entered the study (82% response rate).

We collected the data via self-administered questionnaires, face to face interviews, and clinical examinations to assess the effect of dental caries experience and drug use profile as independent variables on patients’ OHRQoL. Self-administered questionnaires requested information about the participants’ demographic characteristics and their drug use profile based on a standard form which is commonly used as a framework in addiction studies in Iran [16]. Demographic characteristics included patients’ gender, age, education, marital status, employment status, and type of the rehabilitation center (as an indicator of the patients’ economic status). Drug use profile included the main drug of abuse (the most problematic drug which made the patient to ask for treatment), the age at the start of drug abuse, the duration of addiction, and the duration of current treatment.

To calculate a combined indicator for socioeconomic status of the patients, educational level, marital status, employment status, and type of rehabilitation center were dichotomized. For education: 1 = higher education (high school diploma or higher), 0 = lower education (less than high school diploma); for marital status: 1 = married, 0 = single (single, widow or divorced); for employment status: 1 = employed (full-time or part-time), 0 = unemployed (unemployed, retired, homemaker or student); and for type of rehabilitation center: 1 = private, 0 = public. The dichotomized scores were then summed up ranging from 0 to 4 [17].

Personal interview about OHRQoL was conducted by a trained senior dental student using the OIDP instrument. This instrument consists of 11 questions with dichotomous yes or no answers regarding the impact of oral problems on daily life performances: eating, speaking, oral hygiene, doing light physical activities, going out, sleeping, relaxing, smiling, emotional stability, enjoying the contact of other people and carrying out main role or work. The prevalence of OIDP was recorded as the percentage of patients reporting at least one oral impact on their daily performance during the previous 6 months The frequency of each impact (ranging from 1 to 5), the severity of each impact (ranging from 0 to 5), and the specific oral conditions causing the impacts were also assessed. For each individual, OIDP score was calculated as the sum of different performance scores (severity score × frequency score) divided by the maximum possible score, then multiplied by 100 to provide a percentage score ranging from 0 to 100. Validity and reliability of the Persian version of the questionnaire had been confirmed in a previous study on a sample of Iranian population [10].

The same senior dental student was trained and calibrated to carry out clinical oral examinations. Examinations were carried out under the artificial light of a headlamp using disposable dental mirrors and explorers based on the WHO criteria [18] to record DMFT (Decayed, Missing, Filled Teeth), DT (Decayed Teeth), MT (Missing Teeth) and FT (Filled Teeth). Teeth with clinically visible lesions (as cavity or undermined enamel), or when the dental explorer penetrated to the soft dental tissue structures were considered as Decayed Teeth.

### Statistical analysis

We used the Statistical Package for Social Science (SPSS 20.0/PC; SPSS, Chicago, IL, USA). Descriptive data were reported by means (SD) and frequencies. Student’s *t* test, one way analysis of variance, pearson correlation coefficient, and spearman correlation coefficient were used for analysis (level of significance < 0.05). To analyze the factors associated with OIDP among patients with SUD, a linear regression model was fitted to the data.

### Ethical approval

This study was approved by the secretory of the Medical Ethics Committee in the research committee of Islamic Azad University of Isfahan (Research Code: 23810201941035). All volunteer participants provided their informed consent and filled in anonymous questionnaires.

## Results

### Demographic characteristics and addiction profile

Among the patients with SUD, only 1% were women. The mean age of the participants was 55.1 (± 9.9; range 25–75). Most patients were married (61%), were employed (68%), and 42% had diploma or higher education. The mean age at start of drug use was 21.7 ± 6.3. The mean duration of addiction and the mean duration of current treatment were 15 ± 8.6 years and 3 ± 2.4 years respectively. A great majority of the participants reported past use of opium (85%) followed by heroin (42.7%), amphetamines (20.2%), and Crystalline Heroin (19.1%). However, the most frequently reported main drugs were opium (54.7%) and heroin (32.2%). The most common routes of drug use before treatment were combined routes (44.6%) followed by smoking route (36.7%). Only 1.9% reported drug injection (Table 1).

**Table 1:** Demographic characteristics and drug us profile of in-treatment patients with substance use disorders (n = 267)

Variable	N (%)
Gender	
Male	259 (99)
Female	3 (1)
Marital status	
Married	161 (61)
Single	83 (31.1)
Divorced	21 (7.9)
Employment status	
Full time	113 (44.5)
Part time	60 (23.6)
Unemployed	68 (26.8)
Others (student, retired, homemaker)	13 (5.1)
Education	
Illiterate	10 (3.8)
Under diploma	143 (54.2)
Diploma	90 (34)
University education	21 (8)
Main drug	
Opium	146 (54.7)
Heroin	86 (32.2)
Crystalline heroin	12 (4.5)
Amphetamine	7 (2.6)
Other drugs	8 (3)
Unknown	8 (3)
Route of drug administration	
Smoking	98 (36.7)
Snorting	14 (5.2)
Oral	26 (9.7)
Injection	5 (1.9)
Combined method	119 (44.6)
Unknown	5 (1.9)

### Oral health-related quality of life

The mean score of OIDP among patients with SUD attending addiction treatment centers was 22.4 (SD = 8.6). No significant difference existed among dentate (21.9 ± 8.5) and edentulous patients (23.1 ± 8.9) in terms of their OIDP score (*p* > 0.05).

The prevalence of OIDP was 74.1% meaning that as high as three forth of the patients reported at least one oral impact on their daily performance during the previous 6 months. The most prevalent OIDP impact was “difficulty eating” experienced by 64.8% of the participants. Other less prevalent but also considerable impacts included “difficulty showing teeth while smiling”, “higher emotional sensitivity”, “difficulty in enjoying the contact of other people”, and “difficulty in going out” reported by 33.7%, 32.6%, 31.8%, and 30% of them respectively (Table 2).

**Table 2:** Prevalence of oral impacts on daily performances of in-treatment patients with substance use disorders (n = 267)

Daily performance	N (%)	Experiencing any problem with daily activities caused by mouth or teeth in the previous 6 months	
		Regularly N (%)	Partially N (%)
Eating	173 (64.8)	134 (50.2)	39 (14.6)
Speaking	68 (25.5)	65 (24.4)	3 (1.1)
Oral hygiene	11 (4.1)	5 (1.9)	6 (2.2)
Doing light physical activities	34 (12.7)	22 (8.2)	12 (4.5)
Going out	80 (30)	71 (26.6)	9 (3.4)
Sleeping	66 (24.7)	19 (7.1)	47 (17.6)
Relaxing	61 (22.9)	16 (6)	45 (16.9)
Smiling	90 (33.7)	88 (33)	2 (0.7)
Emotional stability	87 (32.6)	76 (28.5)	11 (4.1)
Enjoying the contact of other people	85 (31.8)	75 (28.1)	10 (3.7)
Carrying out main role or work	44 (16.5)	31 (11.6)	13 (4.9)

According to Table 3, the most prevalent cause of the impacts on daily performances were “dental decay” and “tooth loss”, each reported by 62.9% of the participants. Moreover, other prevalent causes were “tooth Fracture”, “Calculus”, “dental pain”, and “tooth colour” reported by 53.6%, 49.8%, 43.8%, and 43.1% of the patients respectively.

**Table 3:** Prevalence of specific oral conditions causing the impacts on daily performances of in-treatment patients with substance use disorders (n = 267)

Oral conditions	N (%)
Sensitive tooth	47 (17.6)
Tooth decay	168 (62.9)
Fractured tooth	143 (53.6)
Tooth loss	168 (62.9)
Mobile tooth	26 (9.7)
Teeth colour	115 (43.1)
Position of teeth (e.g. crooked, protruded, gap…)	2 (0.7)
Size or form of tooth	7 (2.6)
Bleeding of gum	24 (9)
Swollen gum (gum abscess)	11 (4.1)
Receded gums	16 (6)
Calculus	133 (49.8)
Oral ulcer	10 (3.7)
Bad breath	60 (22.5)
Deformity of mouth or face (e.g. cleft in lip, cleft in palate)	5 (1.9)
Clicking in jaw	4 (1.5)
Inappropriate filling (e.g. broken, colour)	10 (3.7)
Mobile or inappropriate denture	38 (14.2)
Orthodontic appliance	1 (0.4)
Tooth surface wearing	25 (9.4)
Tooth pain	117 (43.8)

### Dental health status

The mean score of DMFT among in-treatment patients with SUD was 21.5 (SD 7.2; range 2–28). Missing teeth comprised the major part of the index (mean 15.9; SD 5.03; range 0–28) followed by decayed teeth (mean 4.7; SD 2.4; range 0–17). The mean number of the filled teeth was very low (mean 0.9; SD 0.5; range 0–18).

### Factors associated with OIDP among patients with SUD

The association of demographic characteristics, drug use profile, and dental caries experience with participants’ OIDP has been shown in Tables 4 and 5. In univariate analysis, no significant association revealed between OIDP and patients’ gender (*p* = 0.76), age (*p* = 0.25), years of education (*p* = 0.21), marital status (*p* = 0.74) and employment status (*p* = 0.64). Moreover, no significant association existed between the type of addiction treatment center (private or governmental) and OIDP (*p* = 0.56). In terms of drug use profile, no significant relationship revealed between OIDP and participants’ main drug (*p* = 0.72), age at start of drug use (*p* = 0.33), duration of addiction (*p* = 0.82), and duration of current treatment (*p* = 0.79). In univariate analysis, higher score of OIDP was associated with higher caries experience in terms of DT (r = 0.196, *p* = 0.001) and DMFT (r = 0.155, *p* = 0.01). No significant association existed between OIDP and patients’ MT (*p* = 0.54) and FT (*p* = 0.36).

**Table 4:** OIDP score of in-treatment patients with substance use disorders according to their demographic characteristics and drug use profile (n = 267)

Variable	Categories (N)	Mean (SD)	*p* value
Gender	Male (259)	22.4 (1.5)	0.76
	Female (3)	26.7 (15.2)	
Marital status	Married (161)	22.6 (1.9)	0.74
	Single (83)	21.7 (2.5)	
	Divorced (21)	26.1 (5.4)	
Employment status	Full time (113)	22.04 (2.3)	0.64
	Part time (60)	23 (2.9)	
	Unemployed (68)	23.2 (2.9)	
	Others (student, retired, homemaker) (13)	14.3 (5.9)	
Type of treatment center	Governmental (115)	21.4 (2.2)	0.56
	Private (152)	23.1 (1.9)	
Main drug	Opium (146)	21.8	0.72
	Heroin (86)	24.4	
	Other drugs (27)	23.8

**Table 5:** The association of OIDP among in-treatment patients with substance use disorders with their demographic characteristics, drug use profile, and dental caries experience (n = 267)

Variable	N	r	*p* value
Age	267	0.07	0.25
Years of education	264	0.08	0.21
Age at start of drug use	264	− 0.061	0.33
Duration of addiction	252	− 0.015	0.82
Duration of current treatment	260	− 0.017	0.79
Decayed, missing, filled teeth	265	0.155	0.01
Decayed teeth	265	0.196	0.001
Missing teeth	265	0.038	0.54
Filled teeth	265	− 0.056	0.36

In multivariate analysis, controlling for socio-demographic characteristic and drug use profile, participants with higher DMFT and DT, reported greater oral impact on their daily performance (Table 6).

**Table 6:** Factors associated with OIDP of in-treatment patients with substance use disorders based on a linear regression model (n = 267)

Variable	Beta coefficient	*p* value	95% CI
Age	− 0.03	0.83	− 0.65 to 0.52
Socioeconomic status^a^	0.12	0.09	− 0.42 to 5.73
Main drug (heroin)^b^			
Opium	− 0.03	0.72	− 8.78 to 6.04
Others	− 0.01	0.85	− 12.21 to 10.05
Age at start of drug use	− 0.06	0.50	− 0.79 to 0.38
Duration of addiction	− 0.08	0.49	− 0.81 to 0.39
Duration of current treatment	− 0.08	0.28	− 2.19 to 0.63
Decayed teeth	0.19	0.02	0.15–1.93
Filled teeth	− 0.14	0.06	− 2.91 to 0.03
Decayed, missing, filled teeth	0.34	0.002	0.43–1.92
Dentition status (edentulous)^b^			
Dentate	0.14	0.25	− 4.92 to 18.57

R^2^ = 0.12

^a^Combination of the following variables: education, marital status, employment status, and type of treatment center

^b^Reference group

## Discussion

In order to assess self-perception of oral health among in-treatment patients with SUD, we used the OIDP instrument. Although this is a commonly used and theoretically sound instrument to measure the impacts of oral conditions on individuals’ daily activities [10, 13], other available evidence on OHRQoL among drug dependents have used the Oral Health Impact Profile-14 instrument [5–8]; leading to less data for comparison. The present study revealed at least one impact of oral conditions on daily performance of a majority of the patients. Patients with higher DMFT and DT reported greater oral impact on their daily performances.

Oral impact on daily performance was much greater among patients with SUD in our study (mean 22.4) than among Iranian general population (mean 4.15) [10]. The prevalence score of OIDP in these patients (74.1%) was also higher than that of the general population (64.9%) [10]. This is in line with other similar studies that OHRQoL among patients with SUD was worse than their counterparts in general population [5, 6]. A great majority of the participants (74.1%) reported at least one performance or daily activity affected by oral problems. This finding is similar to that of a sample of Portuguese in-treatment alcoholic patients that as high as 62.4% reported a negative impact on OHRQoL fairly often or often [19]. In addition to a group of homeless people in Brazil with the prevalence score of 81.9% [12], as high as 74.5% of Brazilian crack users reported a negative impact on OHRQoL at least fairly often [7].

In accordance with the result of the study of Iranian general population [10], and the study of homeless people in Brazil [12], the most prevalent OIDP impact during the previous 6 months was “difficulty eating”. The most prevalent oral conditions affecting daily activities were tooth loss and dental decay. Similarly, the most prevalent oral conditions among Brazilian homeless people were need for jaw prosthesis and dental caries [12].

No significant association revealed between OIDP and patients’ demographic characteristics. This is in contrast with the results of the study of a sample of injecting drug users in Australia which poor OHRQoL was associated with female gender, lower education, and unemployment status [6]. Very few female patients with SUD in our sample (1%), might have resulted to the lack of statistical power to detect any gender difference in OIDP. Furthermore, among Brazilian patients with alcohol and substance use disorder, those with lower income were more likely to have poorer general quality of life [4].

Based on the studies either in general population or among patients with SUD, people with higher socioeconomic status in terms of their income, employment, and/or educational level, are less likely to have poorer oral health and poorer quality of life [4, 6, 14, 20]. However, in the present study it seems that other factors might play a more important role in the quality of life of these patients than do their socio-demographic characteristics. This is in line with the results of the study of OHRQoL among drug dependents in South Brazil [8], and the study of Brazilian homeless people, in which socio-demographic factors were not associated with OHRQoL of these patients [12].

No significant association existed between OIDP and participants’ drug use profile. In contrast, several studies have reported poorer quality of life among crack users [4, 7, 8]. This difference in findings might be due to the nature of the drugs. Cocaine/crack was the only addictive drug which showed a significant association with the quality of life of patients in the study by Marques et al. [4]. Based on the report of De Souza, among various substances, only the amount of crack consumed per day was associated with worse impacts [8]. Crack is a street name for cocaine in western countries, and is a highly addictive stimulant, and a popular drug. However, this same street name in Iran mainly contains heroin, with no stimulant effects [21].

Among US methamphetamine users, some aspects of OHRQoL were poorer in moderate and heavy users than in light users [5]. In the study of injecting drug users in Australia, poor OHRQoL was associated with injecting drugs for 10–20 years, and being in methadone treatment [6]. Very few injecting drug users in our sample (1.9%), might have resulted to the lack of statistical power to detect the effect of injecting route of drug administration on quality of life.

Similar to another study of in-treatment patients with SUD in Iran (mean DMFT 20.3) [14], the mean DMFT of patients in our study was high (21.5). Caries experience among our participants was much higher than that of in-treatment patients with alcohol and substance use disorders in Brazil (mean DMFT 13) [4], Southern Brazilian drug users (mean DMFT 11) [8], Brazilian crack users (median DMFT 7) [7] and a group of homeless people in Brazil (mean DMFT 14.4) [12]. Such difference in caries experience between our participants and those of other similar studies seems to be due to the higher mean age of patients in the present study (55.1 years).

Based on our findings, caries experience was a predictor of poor OHRQoL among patients with SUD. This is in line with the results of the study by De Souza et al. that more impacts were reported by those drug dependents with DMFT > 10 [8]. Marques et al. also demonstrated the association between general quality of life of patients with SUD and their caries experience [4]. In the present study, caries experience was the only predictor of poor OHRQoL. Thus, it seems that the effects of drug abuse history on OHRQoL of Iranian drug dependents in our study might have be mediated by their clinical oral conditions. In contrast, among Southern Brazilian drug users, in addition to poor dental health status, the higher amount of crack smoked per day was associated with worse OHRQoL [8]. Furthermore, based on the report of Antoniazzi et al. [7], crack use had a negative effect on OHRQoL independently of demographic backgrounds and oral health status.

Studies on OHRQoL among patients with SUD are scarce. To the best of our knowledge, this is the first study among Iranian patients with SUD in methadone maintenance therapy addressing the issue of OHRQoL. The stratified cluster random sampling method and the high response rate are strength of this study. However, due to the cross-sectional design, it is not possible to infer a causation association. Furthermore, in-treatment patients are not representative of all patients with SUD, thus we cannot generalize the results. Although, OIDP instrument is a commonly used, relatively brief and a theoretically sound measure to assess oral health-related quality of life, the questionnaire nature of the study along with the recall bias due to the report of any problems in daily activities during the previous 6 months are another limitations. However, we tried to increase the validity of the study by objective data collection via clinical examination.

## Conclusion

The present study suggests that there is an oral impact on the daily performance of patients with SUD. Patients with higher dental caries experience reported greater oral impact on their daily performances. Thus, in addition to normative assessment of oral health, clinicians should consider the patients’ self-reported oral problems, and the social and mental aspects of oral conditions.

## Data Availability

The dataset used and/or analyzed during the current study is available from the corresponding author on reasonable request.

## Abbreviations

SUD: Substance use disorders
OIDP: Oral Impacts on Daily Performances
OHRQoL: Oral health-related quality of life
DMFT: Decayed, missing, filled teeth
DT: Decayed teeth
MT: Missing teeth
FT: Filled teeth
